# The Story of the Insane from Year to Year

**Published:** 1902-02-22

**Authors:** 


### THE STORY OF THE INSANE FROM YEAR TO YEAR.

FOURTEENTH ANNUAL SERIES.

### MONMOUTHSHIRE ASYLUM, ABERGAVENNY.

AN increase of 15 patients has to be recorded here during 1900, there being 1,065 in residence on the last day of December. There were 172 first admissions, 31 readmissions, 71 recoveries, 16 discharged relieved, and 88 deaths. The average number resident was 1,050, and the total number of persons under treatment was 1,245. Calculated on the admissions the recoveries stand at 36·5 per cent., and, calculated on the average number resident, the deaths stand at 8·3 per cent. Of the deaths 26 were due to cerebral and spinal diseases, 11 to pneumonia, 13 to consumption, and 9 to other lung diseases. In 23 of the year's admissions the insanity was caused by intemperance in drink, in 40 by hereditary influence, in 31 by previous attacks, and in 14 the disease was congenital. The highest number of patients admitted in any month was in August and the lowest in May. Dr. Glendinning remarks on the small number of deaths caused by general paralysis, and adds that there is a decrease in the number of patients suffering from that disease at present in the asylum, so that the low death rate is not owing to prolonged life of general paralytics. The somewhat high proportion of deaths from lung diseases is not commented on, but we learn that there was no epidemic disease. Alterations and improvements are to be carried out at a total cost of £1,650. These are to include a pathological room. The visitors bear "testimony to the skill, ability, and kindness of Dr. Glendinning and the whole staff." The weekly charge for maintenance is 8s. 2d. per head. Out-county cases are charged 11s. and 14s., private patients 21s. and 30s.

### LEICESTER BOROUGH ASYLUM.

ON January 1st, 1900, this asylum contained 571 patients, and on December 31st, the number had fallen to 565. There were 104 first admissions, 36 readmissions, 52 recoveries, 11 discharged relieved, and 81 deaths. The average number resident during the year was 561, and the total number of persons under treatment was 702. The percentage of recoveries on admissions stands at 37·6, and this is about the average of county asylums, while that of the deaths at 14·4 per cent. of the average number resident is decidedly high, and is owing to an outbreak of influenza. Of the deaths, 12 were caused by cerebral and spinal diseases, 4 by consumption, 6 by other lung diseases, and 33 to influenza and its complications. Of the admissions 23 owed their insanity to various moral causes such as domestic trouble, mental, anxiety, etc., 22 to intemperance in drink, 32 to hereditary influence, 30 to previous attacks, and in 37 there was no cause ascertained. Dr. Finch says that the epidemic of influenza began in April and lasted to the end of July, and during those four months, 119 patients were attacked. Large additions to the asylum were opened in 1900. The new block has been given up to male patients, and the old asylum is now devoted to the female patients The total accommodation is for 855. The committee record their "appreciation of the highly satisfactory manner in which Dr. Finch and the officers of the institution have discharged their respective duties." The charge for maintenance of Leicester patients is 11s. 8d. per head per week. Out-counties pay from 14s. to 15s. 9d.

